# Development of an American Indian Diabetes Education Cultural Supplement: A Qualitative Approach

**DOI:** 10.3389/fpubh.2022.790015

**Published:** 2022-02-08

**Authors:** Jamie Wilson, Cynthia Thomson, Samantha Sabo, Anathea Edleman, Michelle Kahn-John

**Affiliations:** ^1^Mel and Enid Zuckerman College of Public Health, University of Arizona, Tucson, AZ, United States; ^2^Center for Health Equity Research, Northern Arizona University, Flagstaff, AZ, United States; ^3^Diabetes Education and Clinical Education, Tuba City, AZ, United States

**Keywords:** Indigenous knowledge, diabetes, CBPR, American Indians and Alaskan Native, health education, diabetes management, cultural adaptation

## Abstract

**Objective:**

The purpose of this study was to culturally enhance a diabetes education program for Diné (Navajo) community members with Type 2 diabetes. Though the recommendation to culturally adapt health education curricula was meant to improve health education for American Indians and Alaskan Natives (AIANs), it has inadvertently created a “one size fits all” approach. This approach does not properly address the need for tribe-specific cultural health messaging, defined as incorporating cultural elements deemed relevant to the population. Tribe-specific health information and programming, such as integrating Diné worldviews and Indigenous knowledge among Diné people as described here, are essential to creating a culturally relevant and effective and meaningful approach to disease self-management.

**Methods:**

A conversation guide, based on the Hózhó Resilience Model—a Diné framework on healthy living, was used to engage key cultural experts in interviews about traditional stories and teachings regarding health and wellness. Three specific self-care behaviors relevant to Type 2 diabetes self-management were discussed: (1) healthy eating, (2) physical activity, and (3) healthy coping. Interviews were audio-recorded, transcribed and analyzed using a qualitative thematic analysis method.

**Results:**

Diné healers and cultural experts informed the development of an educational tool called Diné Health. Key themes that emerged from the data included the importance of discipline, positivity and mindfulness in the context of Hózhó.

**Conclusion:**

Culturally safe and meaningful engagement with cultural leaders and the use of qualitative research methods can inform deep-level cultural adaptations essential to developing tribe-specific diabetes education programs. The approaches used here can guide the development, implementation, and testing of culturally-informed health education for AIAN populations.

## Introduction

Although diabetes prevention and control efforts have increased in American Indian and Alaskan Native (AIAN) communities, AIAN people continue to experience disproportionality higher rates of diabetes-related morbidity and mortality (1). Diabetes is the fourth leading cause of death for the Navajo Nation (2), and is the seventh leading cause of death for the United States in general (US). The age-adjusted diabetes mortality rate for the Diné (Navajo) population is more than double (2.29 times higher) the US population rate (3). Poor glucose management may lead to diabetes complications and premature death, including vision loss, kidney failure, heart disease, limb amputation, and death (3–5). Promising approaches to diabetes management include nationally recognized guidance for self-care, medication management, diabetes education, regular check-ups, and ongoing support (6). The purpose of this study was to culturally adapt a standard diabetes education program originally designed to broadly address DM education needs for AIAN populations collectively. This cultural adapted program is designed specifically for Diné community members diagnosed with type 2 diabetes.

Cultural adaptation has been defined broadly as a modification of an evidence-based intervention by changing the curriculum's language and context in such a way that it is compatible with the client's cultural patterns, meanings, and values (7). To some, cultural adaptation is achieved by making surface-level adaptation such as changing the visual content or by adding AIAN created artwork (8). Deep-level cultural adaptation, on the other hand, has deeper levels of meaningfulness and includes adapting the language, metaphors, content, concepts, goals, methods, and framework to meet the needs of Indigenous communities (8). To optimize the impact of diabetes education for AIAN populations, deep-level cultural adaptations must be considered (9).

In 1997, in an effort to provide culturally competent health programs, Congress established the Special Diabetes Program for Indians (SDPI) to treat and prevent diabetes in AIAN communities (10). Currently 404 Indian Health Service (IHS), tribal and urban Indian health programs across the US receive SDPI funding (10). SDPI grantees recognize that culture and health are intertwined and inseparable concepts. They propose culturally adapted interventions are more acceptable, better understood, and more effective (11). For example, SDPI interventions that include AIAN language, traditional food demonstrations, and cultural activities (11) have demonstrated reduction in diabetes complications such as amputations and kidney failure (10, 12).

Despite well-intentioned efforts to culturally adapt and tailor health education curricula, diabetes programs have inadvertently created a “one size fits all” adaptation approach to health education programs (13) that is commonly used to prevent and manage diabetes among AIAN communities (14). Emergent research, however, suggests that AIAN community members prefer tailored and culturally adapted diabetes education programs that reflect their own cultures. The Native American Diabetes Project (NADP) conducted a participant satisfaction questionnaire regarding cultural competency with eight tribal communities (15). Results revealed that community input in co-developing the diabetes education sessions was an important factor in participant satisfaction and retention in the diabetes education series (15). Griffin et al. (15) highlighted that storytelling, a traditional communication strategy, was recommended by participants as a way to communicate information and provide diabetes education (15). Moreover, participants suggested that more culturally specific components, such as traditional foods, teachings and games could enhance participant satisfaction with educational sessions (15). Roubideaux found that 95% of participants preferred diabetes education materials relevant to their specific tribe or culture (16).

Several studies have shown a high prevalence (16.5%) of diabetes among the Diné (17) and 22.9% for adults (18) and an incidence rate of 2.78 per 1,000 for youth (19). Studies implicate the need for effective culturally relevant education for the Diné (20, 21) however, none of the prior diabetes prevention projects included tailored, deep-level, tribe-specific health information or cultural enhancement of diabetes education that were designed specifically for Diné communities. Efforts to apply surface-level cultural adaptations to the standard AIAN diabetes education curriculum may further perpetuate high program drop-out and may have limited effectiveness in promoting optimal diabetes self-management or glucose control in Diné communities. The purpose of this research was to use a community-based participatory research (CBPR) approach to engage Diné cultural experts and healers, to gain insights about cultural world views and Indigenous knowledge for the purpose of adapting an evidence-based diabetes management intervention for Diné adults diagnosed with Type 2 diabetes.

## Methods

### Research Design

This study used a CBPR approach (22) to engage qualitative research methods and guide the collaborative production of a cultural tool, herein called Diné Health (DH), to be used in diabetes education classes in a Diné community. The lead author, a Diné researcher, met with members of the Diabetes Program a year prior to seeking approval from University and Tribal institutional review boards before engaging in the study. The director of the program helped obtained letters of support for the study from relevant tribal leaders and organization and also co-presented at meetings such as the Navajo Nation Human Research Review Board (NNHRRB) meeting to inform key tribal stakeholders about the study aims and objectives. The lead author applied Indigenous health research practices and Diné values of k'é (i.e., personal conduct, traditional etiquette to establish kinship) to establish respect and build positive relationships with Diné leaders and community members (23, 24). The NNHRRB and the University of Arizona Institutional Review Board approved this study in August 2018. The data and findings from this study belong to the Navajo Nation.

### Community Engagement

The lead author collaborated with community partners to protect and properly apply only approved aspects of sacred Indigenous knowledge of the Diné (Table 1). When working with AIAN communities, it is important to recognize the aspects of protected and private cultural and traditional knowledge. Seeking consultation from cultural experts provides the opportunity to ensure that the research approach is ethical, culturally sensitive and does not disrespect, exploit or misinterpret cultural or traditional worldviews. It is also important to maintain the privacy of the more sacred traditional and cultural knowledge such as traditional stories or ceremonial knowledge which may be restricted to be known only by traditional healers. Some AIAN communities prohibit the recording of traditional knowledge so, it is important to seek the advice of cultural experts to safeguard the interpretation of research findings and to ensure the research approach is acceptable and respectful. Establishing good rapport, including reciprocal learning between researcher and partner, with the diabetes program before the study began was an important first step in this study and in line with CBPR approach (22, 25). The purpose of the initial meeting (held in January of 2018) with the Diabetes Program director was to determine whether the program would like to collaborate in a research study. The program expressed a concern that there was minimal Diné culture in their existing diabetes education classes and only one AIAN health educator who offered classes in the Diné language one time per month. At the time of this study implementation, the diabetes program was in the process of curriculum development. Therefore, the curriculum adaptation proposed in this study presented an opportunity to contribute to the development of a meaningful and sustainable product for the diabetes program.

**Table 1:** Community partnerships and roles in the Diné Health (DH) study.

**Members**	**Role(s)**
Diabetes Program	• Program director helped gain approval for study • Review DH drafts and materials monthly or as needed • Received training on DH • Implement DH into diabetes education classes
Community Advisory Board	• Attend CAB meetings and/or provide feedback via email • Reviewed transcript excerpts from key informant interviews • Approved cultural teachings and messages to be included in the DH
Diné Health Working Group	• Reviewed transcript excerpts from key informant interviews • Selected approved cultural messages to be included in DH classes • Reviewed DH drafts

The lead author purposefully sought guidance and consultation from a Community Advisory Board (CAB) established to support the current programming efforts. The CAB members included community and organizational representatives selected by the community for their professional, cultural and local knowledge. Inclusion criteria for the CAB were: must be an (18 years of age or older), must be a member of the Diné Nation, must possess expertise in Diné culture or Diné health, and Diné ceremony/healing. The CAB served to ensure that the research study respected the Diné people and culture. Lastly, a working group was established to lend expertise and experience in curriculum development. The working group consisted of three diabetes educators from the program and the lead author and met 5 times over 6 months.

### Theoretical Framework

This study used the Hózhó Resilience Model (HRM) (26) as a framework to guide this study. The HRM is based on the Diné Philosophy of Hózhó—the ultimate state of health and wellness (27). Hózhó provides specific rules for a Diné person's behavior and emphasizes order, balance, and harmony in everyday life (26). The importance of order is reflected in a constant mindfulness and reverence with every thought, every word, and every step made in the journey of life (28). The concepts of balance and harmony are parallel to the notion of holistic health which emphasizes that there is a connection between the mind, body and spirit of an individual (23, 24, 29). For the Diné, there is an additional relationship with living elements i.e., water, air, earth and the cosmos (28). This relationship with the elements is driven by respect, specifically respect for plants, animals, and water. For this study, we used the HRM domains and attributes (Table 2) to identify key cultural concepts of the Diné health protective traditional teachings that can influence the self-care behaviors of our DH participants.

**Table 2:** Hózhó Resilience Model domains and attributes.

**Domains**	**Attributes**
Harmony (External factors)	Thinking–Remain positive in thought by practicing mindfulness
Respect (Internal factors)	Discipline–Remain respectful in all actions by having self-discipline
Spirituality (Existential factors)	Spirituality–Remain positive and harmonious through prayer

### Balance Your Life With Diabetes Curriculum

The Balance Your Life with Diabetes (BYLD) (11) curriculum focuses on Type 2 diabetes and self-care and is the standard curriculum used in diabetes programs across Indian Country, including the program in this study (10). The BYLD curriculum was culturally adapted by the IHS Division of Diabetes Treatment and Prevention program. The IHS encourages programs to adapt their program's BYLD curriculum to reflect their participants culture, e.g., traditional food recipes, language, activities and traditional teachings (11). In some cases, however, program staff may not be familiar with the community's culture and therefore have difficulty offering tribe-specific adaptations to the BYLD. To close this gap in knowledge, the study investigators partnered with a diabetes program with a goal to adapt their BYLD curriculum by adding Diné-specific, meaningful and relevant cultural teachings to the BYLD lessons: healthy eating, being active, and healthy coping (Table 3).

**Table 3:** Balance your life with diabetes (BYLD) and Diné Health (DH) supplemental curriculum content.

**BYLD curriculum^a^**	**DH supplemental curriculum^b^**
Lesson: Healthy Eating Knowledge • Effect of foods/beverages on metabolic parameters (including blood glucose, lipids, blood pressure, weight, etc) • Sources and distribution of nutrients (nutrient-dense carbohydrates, lean proteins, healthy fats) • Eating patterns (frequency of meals, timing, portions, etc) • Resources to assist in food ∙ choices ∙ • Macronutrient composition (quality, quantity, combination, substitutions) Skills • Meal planning • Portion awareness and management • Planning strategies (Carb Counting, Exchanges, Plate Method, Mindful Eating) • Nutrition Facts Label comprehension • Special situations and problem solving (planning, shopping, meal delivery/kits, eating away from home at work/school/ restaurants) Barriers • Environmental factors • Cultural and family influences • Food and health beliefs • Financial (food security) • Cognitive • Health literacy and numeracy • Emotional • Meal pattern sustainability	Supplemental Topic: Diné Food Practices Health Message(s) • T'óó bikiinígo (translation- eating for sustenance and nourishment) • Practicing portion control through self-discipline and mindfulness PowerPoint Presentation • Audio Recording: Practicing mindfulness during food preparation, cooking, serving and eating Supplemental Material(s) • DH Mindfulness Worksheet • DH Homemade Food and Meal Preparation Worksheet
Lesson: Being Active Knowledge • Planned exercise (type, duration, intensity, frequency, progression) • Daily movement • Breaking up sedentary time • Safety precautions, such as obtaining preparticipation medical clearance and/or exercise stress testing prior to unaccustomed vigorous activity • Special considerations, such as appropriate footwear Skills • Appropriate daily movement and physical activity plan • Adjustment of activity with food and medication to maintain glycemic balance • Monitoring of cardiometabolic parameters, data stream, and feedback Barriers • Physical (health conditions, injuries) • Perceived lack of time • Environment, facilities • Fear (hypoglycemia) • Self-efficacy • Lack of enjoyment • Lack of social support	Supplemental Topic: Establishing Self-discipline Health Message(s) • Diné teachings on having strong self-discipline in daily activities that foster good health physically and mentally PowerPoint Presentation • Audio Recording: T'aa hwo ajiteego (translation - be self-reliant and proactive) in your health Supplemental Material(s) • DH Discipline Worksheet • DH Daily Routines Worksheet
Lesson: Healthy Coping Knowledge • Internal and external motivators • Benefits of solution-focused problem solving • Active self-management • Value of nurturing support system (peers, online, family, friends) • Individual empowerment • Role as partner with other members of health care team Skills • Goal setting • Problem solving • Coping strategies • Self-efficacy Barriers • Physical	Supplemental Topic: Positive Thinking Health Message(s) • Diné teachings on carrying oneself in a mindful, respectful and positive manner in all aspects of life, especially through one's actions and thoughts PowerPoint Presentation • Audio Recording: Holistic Health and Sa̧'áh Naagháí Bik'eh Hózhóo (Living a long healthy life) Supplemental Material(s) • DH Goal Setting Worksheet
• Financial • Emotional • Competing priorities • Lack of support network • Psychosocial distress including diabetes distress • Cognitive including mental health disorders	

a *Diabetes Self-Management Education and Support (DSMES) Core Outcome Measures (6)*.

b *Diné-specific cultural teachings/topics added to the BYLD Group Classes*.

### Cultural Expert Participation Selection and Interviews

Through a purposive sampling strategy, the lead author worked with leadership of a Diné healer association to identify well-respected healers and cultural experts who hold firsthand knowledge about the Diné traditional teachings and culture. The interview participants included five (n = 5) tribal members that spoke Diné and English, were distinguished healers within their respective communities and each resided on the Navajo Nation. The interviews were 60–90 min long and were conducted in a location most convenient to the healer, either at a central location or the healer's home. Data from the interviews guided the development of a tailored, tribe-specific educational tool for diabetes education—herein called Diné Health (DH). Each participant was provided a copy of the DH at the completion of the study. All participants provided informed consent and received an incentive to cover direct and indirect costs associated with participation in the study.

### Interviews

Five in-depth interviews were conducted to elicit cultural health messages from the aforementioned Diné cultural experts. With consultation from a Diné researcher, the lead author developed a structured conversation guide (eight questions) to gather culturally grounded health messages regarding Hózhó. The questions asked about three diabetes self-management and education (DSME) self-care behaviors: (1) healthy eating, (2) physical activity and (3) healthy coping (6). Four questions focused on traditional food ways, self-discipline and mindfulness, e.g., “Based on Hózhó, what stories and teachings do you know about traditional food ways?” Two questions asked about physical activity and self-discipline, e.g., “Based on Hózhó, what stories and teachings do you know about being disciplined in physical activity?” The last two questions were asked about the importance of positive thinking and health, e.g., “Based on Hózhó, what stories and teachings do you know about thinking positively? As Diné, how do we show our respect to food and our health?” The CAB and diabetes working group reviewed the questions. The interviewer—a trained community researcher—is Diné who speaks, writes and understands the Diné language. Data from the interviews were audio recorded, transcribed verbatim and analyzed using a qualitative thematic analysis method.

### Analysis

The lead author translated and transcribed audio recordings from Diné to English. To ensure accuracy and context, a consultant, who is Diné and holds cultural and linguistic knowledge, reviewed each of the transcripts. Although time-intensive, this process ensured complete and accurate transcripts. The lead author conducted deductive thematic analysis (30), driven by the researcher's theoretical interest in the context of the HRM and DSME. The aforementioned HRM domains served as the theme categories: harmony, respect and spirituality. The lead author read all transcripts independently while highlighting repetitive words and phrases which were coded as patterns. As a secondary step to review cultural alignment and interpretation and application of the HRM attributes in the DH, the lead study author also reviewed themes with the HRM developer. A thematic matrix was developed in Excel to organize and compare the themes and patterns from each transcript. This process helped the lead author identify common emergent themes and patterns. Once saturation -no new data—was reached, the lead author entered quotes into the Excel matrix. The themes identified in this by responses provided by the Diné cultural experts and healers were used to inform and guide the development of the DH (see Table 4).

**Table 4:** Themes from key informant interviews.

**Themes**	**Patterns**
Relationship with food, family and culture	• Traditional food connects the Diné people to Mother Earth • T'óó bikiinígo (eat for sustenance and nourishment) • Diné custom to cook for all relatives • Having good thoughts while cooking ceremonial food
Self-discipline is needed for good health	• Engaging in physical activity shows self-discipline and strength • T'aa hwo ajiteego (being self-reliant and proactive) • Laziness is considered taboo in the Diné culture
Positive thinking is connected to health	• Thoughts can affect one's health • Belief in a holistic connection of Diné people's mind, body, spirit to Mother Earth and all living things • Mindfulness is needed in all aspects of life

## Results

Four Diné male healers and one female cultural expert were interviewed for a total of five cultural expert and/or healer participants. Four participants were Diné healers knowledgeable about cultural teachings. One participant did not identify as a healer but was affiliated with the healer association as a cultural expert. Key themes from the data included the importance of the concepts and behaviors of discipline, positivity, and mindfulness—each attribute of Hózhó as outlined in the HRM, which were identified to be important aspects of wellness behaviors. When asked about health, participants discussed traditional Diné teachings and cultural activities that promote holistic health.

Participants also spoke about the importance and fundamental teachings of living in Hózhó, the ultimate state of health and wellness for the Diné. One healer explained, “As Diné we have a purpose for everything we do, so…we should think about what we put in our bodies and how we treat our bodies. How we think about things is important when we talk about Hózhó.”

Another healer said,

Hózhó means more than harmony, it is our whole life. We, the Diné, know what it means to live life in a good way. We've been instructed by our Holy People how to accomplish this…it requires a lot of hard work and discipline. It means to be healthy, happy, humble and be in harmony or balance with everything we encounter.

Three reoccurring culturally informed health promotion and self-management themes arose from the transcripts and were used to guide the development of DH:

Theme 1: *T'óó bikiin*í*go* (translation—eating just enough). Some messages were central to healthy eating, e.g., being mindful of portion sizes is important to prevent overindulgence and wastefulness. Overindulgence is not the traditional way of eating for the Diné. One healer said,

*T'óó bikiin*í*go* (translation—I eat just enough to get by, not too much). We eat food when we are hungry, not when we are craving something. We shouldn't eat too much, we should eat just enough to sustain our bodies.

Wasting traditional food is disrespectful to the Diné people, it shows poor values. A healer explained,

Ceremonial food should not be wasted, doing so is considered disrespectful to the plants, the Earth and to our mother, Changing Woman, [a holy deity]. She [Changing Woman] put food here [on Earth] for us, her children. She left us teachings to live life in a respectful way, not to do things without having a purpose.

Further, participants discussed key practices around food preparation were discussed (e.g., praying and having good thoughts while cooking). “Eating food that has been made by the fire in a hogan is the best medicine, because homemade chiyaan (food) is made with prayer and good thoughts…so when you eat it, you should feel better,” said a Diné healer.

Theme 2: *T'aa hwo ajiteego*. (Translation—It is up to you to accomplish things in your life, no one is going to do it for you). For the Diné people, self-discipline is needed in every aspect of life from dawn to dusk (28). One strict disciplinary teaching is to offer a morning prayer and run in the east direction. The Diné believe that when a person adheres to this teaching, the individual will be blessed with a good life, including good health and wellness. A Diné healer said,

If you are disciplined, you won't be lazy. This is *t'aa hwo ajiteego*. If you put your mind to something, you have to do it, it is up to you. We must have this type of mindset to have good things in life. This is the teaching of Hózhó.

All participants emphasized that this teaching is essential to living in Hózhó; self-discipline is needed to establish good physical, mental, and spiritual health.

Theme 3: Role of mindfulness is fundamental to health and wellbeing in the Diné culture. The Diné are told to have strong respect of the self through discipline and mindfulness (28). For the Diné, mindfulness means that all actions must have a purpose. They believe that the Holy People are watching over them, therefore they must carry themselves in a mindful, positive and respectful manner. Negative thoughts and behaviors are strongly discouraged. Doing so brings disharmony and imbalance. A healer explained,

Ada *akooznizin'go nijigha* (translation—be mindful, have self–awareness and respect). Our people [the Diné] believe that a disciplined and positive mind keeps individuals from inviting *Té'i'i* (translation—poverty) into their lives. It [positive thinking] shields us from the negative things in life, it is our protection. Having this kind of mindset is Hózhó, so I encourage people to keep striving and you will get there.

Healers also explained the Diné belief in the holistic connection of the mind, body, spirit to Mother Earth and all living things i.e., plants, animals, the elements.

### Program Adaptation

The DH working group met four times to review the findings from the study (Table 3) and helped select key health messages to be included in the diabetes education class. The lead author developed four DH worksheets based on the group's selections. The DH worksheets were used as in-class handouts to supplement the BYLD PowerPoint, with the goal of adding more discussion around Diné culture and resilience to the BYLD. The CAB approved all DH materials with minor edits, e.g., Diné spelling and grammar.

The lead author provided a 1-day training on the DH supplemental curriculum for the Diabetes Program staff. This training took place 1 month prior to the implementation of the DH. The lead author attended the first few classes to observe and take notes on the implementation of the DH. The lead author used an observational checklist to identify the limits of the DH. Based on the lead author's notes and feedback from the health educators, several gaps to the DH were identified, such as the difficulty of reading the content written in the Diné language and the need for more cultural context behind the health messages. The lead author audio recorded all words, phrases and health messages written in the Diné language. The audio clips were embedded into the PowerPoint presentations. Sample Diné phrases that were audio recorded include: (1) *T'óó bikiin*í*go*, meaning to eat just enough to live and not to overindulge in food consumption and (2) *T'aa hwo ajiteego*, meaning a person must rely on their own will and determination to achieve their goals. The DH was developed with the goal of utilizing culturally grounded teachings to teach patients with diabetes about diabetes self-care and management, thus enhancing the cultural relevancy of the current programming.

## Discussion

A key strategy to increasing diabetes education attendance and retention rates for AIANs is to implement culturally grounded, tribe-specific curriculums into community-based health programs (10). The main findings of this study include (1) CBPR approaches are feasible to inform development and adaptation of health programs in AIAN communities and (2) Indigenous health research practices and qualitative methods can be applied to strengthen existing curriculum, as in this case, in the form of the DH supplemental curriculum.

Researchers used CBPR to ensure that the results of the study can be used for action and sustained by the community (22, 25). In this study, several steps were essential to ensure that the Diabetes Program could implement and sustain the DH. First, the lead author established a relationship with the program before writing the study proposal. Establishing a reciprocal relationship with the community early in research is important in CBPR, especially in tribal communities. The diabetes program staff took initiative in all phases of the study by writing letters of support, attending leadership meetings, helping obtain IRB approvals from the university and tribe and co-developing the curriculum. Second, the lead author applied Indigenous health research practices and Diné values of k'é (i.e., personal conduct and kinship) to establish respect and build positive relationships with Diné leaders and community members (23, 24). Second, in addition to the program's contribution in the curriculum development, other key stakeholders, such as cultural experts and healers were actively involved. The integration of the Diné healers' and cultural expert's Indigenous knowledge certified and validated the cultural rigor that was used to inform the adapted DH content. This process used in the development of the DH supplement curriculum exemplifies consideration and respect for the integration and inclusion of Indigenous knowledge for the purpose of gathering information in a culturally appropriate and meaningful way (31).

Utilizing a qualitative approach to develop and inform a cultural curriculum is important for identifying health-protective and strength-based wisdom in AIAN communities. A qualitative voice enriches the curriculum and adds depth, richness, and meaning to the educational content. The Native American Diabetes Project (NADP) also used a qualitative approach to design culturally relevant education materials. In the NAPD, the community was an integral part of the project; they participated in the interviews, focus groups and community advisory board (32). A unique feature of our study was the rich cultural context and experience tribal scholars brought to the table. The Diné cultural experts' and lead author's cultural knowledge were imperative to understanding the depth and strength of the health messages. The qualitative approaches used in this study can guide the development and implementation of culturally-based, tribe-specific health education model tailored for AIAN populations, which can then be implemented for the Diné individuals diagnosed and living with diabetes. Outcomes of culturally informed and adapted health promotion programs should be studied further for outcomes and effectiveness within each tribal community that receives an adapted curriculum.

The study has a few limitations. First, there were only a handful of key informant interviews conducted due to time constraints and competing commitments such as work, expectations of both professional careers, and/or commitments to patient appointments for healing ceremonies. Despite the recruitment of five participants (n = 5), however, data saturation was achieved. Second, only one female participated in the key informant interviews. The Diné people are a matrilineal culture, therefore more insight from female healers could add to the curriculum, especially in regard to traditional food ways and healthy eating. Third, the community partners were not initially involved in the analysis of data, due to the training required to analyze qualitative data. In the hopes of mediating this limitation, the CAB and DH (many with expert cultural knowledge) working groups played vital roles in identifying culturally appropriate health messages for the curriculum.

A significant strength in this project was how the collective wealth and richness of authentic cultural knowledge shared by healers, CAB members and the diabetes program team members contributed to the study. Their willingness to share, learn and collaborate with the lead author on this study required additional work and time, but their dedication to properly honoring Indigenous health research was integral to developing a culturally grounded curriculum. This study could be used to guide the development and implementation of other culturally-based and tribe-specific health interventions for AIAN populations. Most important, the robustly tailored program will need to be tested to determine the impact on program enrollment, engagement, recidivism, and clinically relevant health outcomes such as diabetes self-management and glucose control. Lastly, the DH is not meant to serve as an independent diabetes curriculum, it is a supplemental curriculum that should be used in conjunction with a standard diabetes education curriculum specifically for the Diné people. The CAB members felt strongly about keeping and integrating the DH supplement in the existing DM program because it added authenticity and meaningfulness that countered the existing predominantly western based or mainstream diabetes education program.

## Conclusion

Culturally safe and meaningful engagement with cultural leaders, along with the use of qualitative research methods can inform deep-level cultural adaptations essential to developing robust diabetes education programs at the individual tribe level. Such work can illuminate cultural relevance and may be important in identifying culturally related factors that influence engagement and completion as well as health outcomes for participants in AIAN diabetes education programs. We recommend qualitative CBPR approaches and Indigenous research practices used here to guide the development and implementation of culturally tailored tribe-specific educational model that has been informed by the Diné cultural teachings.

## Data Availability Statement

The datasets presented in this article are not readily available because the data belongs to the Navajo Nation, according to the Navajo Research Act and longstanding IRB policy.

## Ethics Statement

The studies involving human participants were reviewed and approved by Navajo Nation Human Research Review Board. The patients/participants provided their written informed consent to participate in this study.

## Author Contributions

JW wrote the manuscript and made revisions to manuscript as part of her dissertation. CT, MK-J and SS provided comments on drafts and helped with final edits. All authors contributed to the article and approved the submitted version.

## Conflict of Interest

Author AE was employed by Tuba City Regional Health Care Corporation. The remaining authors declare that the research was conducted in the absence of any commercial or financial relationships that could be construed as a potential conflict of interest.

## Publisher's Note

All claims expressed in this article are solely those of the authors and do not necessarily represent those of their affiliated organizations, or those of the publisher, the editors and the reviewers. Any product that may be evaluated in this article, or claim that may be made by its manufacturer, is not guaranteed or endorsed by the publisher.
